# Supramolecular Assembly of pH-Sensitive Triphenylene Derived π-Gelators and Their Application as Molecular Template for the Preparation of Silica Nanotubes

**DOI:** 10.3390/gels2010007

**Published:** 2016-02-01

**Authors:** Ignacio Muñoz Resta, Verónica E. Manzano, Florencia Cecchi, Carla C. Spagnuolo, Fabio D. Cukiernik, Pablo H. Di Chenna

**Affiliations:** 1 UMYMFOR-CONICET, Departamento de Química Orgánica, Facultad de Ciencias Exactas y Naturales, Universidad de Buenos Aires, pabellón 2, C. Universitaria, CABA, C1428EGA Buenos Aires, Argentina; imresta@qo.fcen.uba.ar; 2 INQUIMAE, Departamento de Química Inorgánica, Analítica y Química Física, Facultad de Ciencias Exactas y Naturales, Universidad de Buenos Aires, pabellón 2, C. Universitaria, CABA, C1428EGA Buenos Aires, Argentina; veromanz@qo.fcen.uba.ar (V.E.M.); flo_cecchi@yahoo.com (F.C.); fabioc@qi.fcen.uba.ar (F.D.C.); 3 CIHIDECAR-CONICET, Departamento de Química Orgánica, Facultad de Ciencias Exactas y Naturales, Universidad de Buenos Aires, pabellón 2, C. Universitaria, CABA, C1428EGA Buenos Aires, Argentina; carlacs@qo.fcen.uba.ar

**Keywords:** pH-sensitive, triphenylene, π-gelators, functional gels, organogels, supramolecular gels

## Abstract

The gelation properties and mode of self-assembly of six asymmetrical hexaether triphenylene derivatives mono-functionalized with carboxylic and primary amine groups were investigated. The presence of a carboxylic and amine group attached to the triphenylene core generated stable, thermo- and pH-sensitive supramolecular π-organogels with a reversible response to both stimuli. In order to understand the gelation process, we studied the effect of the spacer length and found a different gelation scope for the acid and basic derivatives that accounts for a different supramolecular self-assembly. The presence of the basic group on the amino derivatives was used to guide and catalyze the templated *in situ* sol-gel polymerization of TEOS and allowed us, under controlled hydrolytic conditions, to prepare an entangled fibrillar network of silica nanotubes.

## 1. Introduction

Supramolecular gels are unique colloidal soft materials built up by the self-assembly of small molecules (low molecular weight gelators, LMWG) in a given solvent through the formation of a 3D network. Due to the reversible nature of the non-covalent supramolecular interactions that held the system together (hydrogen bond, ion-ion and dipole-dipole interactions, π-π stacking, *etc*.) supramolecular gels are thermosensitive and can be transformed reversibly to a fluid (sol) by heating above the gel-sol transition temperature (T_gel_), and *vice versa*. Moreover, supramolecular gels have the chemical and physical functional properties inherent to the gelator molecules and, if the functionality can be modulated externally, the gels become sensitive to chemical [1] or physical stimuli [2], such as pH, ions, light, *etc.* This unique characteristic makes of every supramolecular gel system a potential functional soft material with application in a wide variety of technological areas: from bio-medicine to electronic devices and/or media for chemical reactions [3], sensing [4], self-healing materials [5], molecular template for preparation of inorganic nanoparticles [6], and many others [7]. Among the wide variety of structurally diverse families of LMWG (e.g., peptides, steroids, saccharides, nucleobases, dendrimers, *etc.*) gelators based on π-systems with more than one fused or conjugated aromatic ring, so called π-gelators, are of special interest due to their intrinsic electronic properties derived from the delocalized π-electron system such as luminescence, charge carrier mobility, and electronic conductivity [8]. Therefore, π-gels are promising soft materials with potential application in imaging, sensing and in high-tech organic electronic devices such as LEDs [9], FETs and PVDs [10,11]. Triphenylene derivatives, with a flat four fused ring π-core, are a well-known family of discotic liquid crystals (DLC) that form columnar assemblies with π-stacked aromatic cores surrounded by alkyl chains [12] leading to anisotropic carrier transport materials with higher mobility than conventional semiconductors [13,14]. In triphenylene based DLC the distance between rings is about 3.5 Å with a column separation of 20–40 Å depending on the length of the side alkyl chains. Some liquid-crystalline supramolecular gels composites derived from triphenylene DLC and LMWGs have been developed in order to obtain functional materials with modulated electro-optical and conductive properties [15,16]. Furthermore, some triphenylene derivatives were reported to form π-organogels by themselves due to, for example, cooperative intermolecular hydrogen bonding stabilization of the columnar organization generating Discotic Liquid Crystal Gels (DLCG) [17]. In this area Shinkai and co-workers [18] reported the first example of a symmetric triphenylene gelator substituted with six amide groups that exhibit unusual emission properties from an excimer formation, but does not form DLC phases in bulk. Recently, there have been only two reports of simple mono-functionalized asymmetric triphenylenes DLCG containing imidazole [19] and alcohol [20] moieties linked through spacers to the triphenylene core.

**Figure 1:**
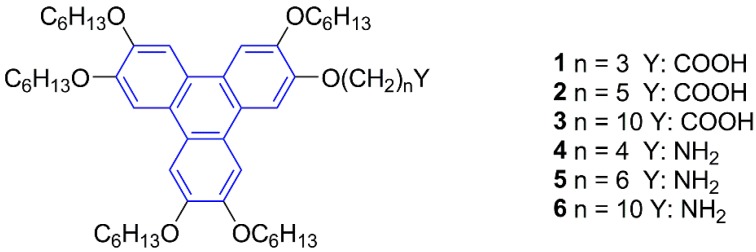
Structures of triphenylene derivatives **1**–**6**. The triphenylene core is shown in blue.

In this work we report on the supramolecular organogelling properties of six asymmetrical hexaether derivatives of triphenylene mono-functionalized with carboxylic and primary amine groups (Figure 1). Although compounds **2**–**6** have already been synthesized and characterized as discotic liquid crystals [21,22,23], and extensively used as intermediates in the synthesis of more complex triphenylene derivatives, their gelling ability has not been reported so far. We demonstrate below that the presence of a carboxylic acid (COOH) or amine (NH_2_) group attached to the triphenylene core generates stable, pH-sensitive supramolecular π-organogels. In order to understand the gelation process we studied the effect of the spacer length finding a different gelation scope for the acid and basic derivatives accounting for a different self-assembly process. Under self-catalyzed conditions, the fibrillar superstructure of the gel of the amine derivative **5** was successfully used as template for the *in situ* sol-gel polymerization of tetraethoxysilane (TEOS) and the further preparation of silica nanotubes.

## 2. Results and Discussion

### 2.1. Gelation Scope and Thermal Stability

Our study started with triphenylene derivative **2**, which was synthesized following reported literature procedures [21]. This acid was able to gel methanol and other small alcohols at 5 °C, generating stable, turbid gels. This simple mono-acid was reported and characterized in several works as an intermediate in the synthesis of other more complex triphenylene derivatives but, to the best of our knowledge, the gelling property has not been reported so far. There is only one example in the literature of a mono-substituted triphenylene π-gelator that bears a primary alcohol with similar gelation ability [20]. In these triphenylene alcohols the self-assembly was proposed to be driven by the π-π interactions between the triphenylene cores with solvation of the OH groups in the periphery. To study the structure/gelling property relationship of the carboxylic derivatives we synthesized the series of triphenylene acids **1**–**3** with spacers of different length [21,22], we chose alkyl chains of 3, 5, and 10 carbon atoms in order to have the COOH functionality inside, on the edge and outside the hydrophobic corona generated by the other five hexyloxy substituents. We also included in our study the triphenylene derivatives **4**–**6** with a primary amine functionality. These triphenylene amines were reported to form columnar mesophases when cooled from the isotropic liquid phase with a tendency to lead to glassy structures [23]. This behavior caught our attention on these amines as good supramolecular π-gelators candidates. The gelation scope of compounds **1**–**6** was studied with the simple test tube method over a wide range of organic solvents (Table 1). When no gelation was observed after resting the tube at room temperature it was cooled in the fridge at 5 °C. In general, the three carboxylic acids were insoluble in extreme polarity solvents such *n*-hexane and water (entries 1 and 7) and soluble in middle polar solvents such as ether, ethyl acetate and dichloromethane. Although none of the acids gelled methanol at room temperature, in all cases cooling at 5 °C induced a fast gelation. They also gelled other small primary alcohols such as ethanol and *n*-butanol. To evaluate the effect of the alkyl group of the alcohol on the thermal stability, the gel-sol transition temperature (T_gel_) for gels of compound **2** in methanol, ethanol and *n*-butanol were determined by the inverted tube method at the same concentration of gelator. Analysis of the T_gel_ and the dielectric constants (*ε*) of the alcohols clearly showed that as the *ε* increases so does the T_gel_ accounting for higher thermal stability (Table 2). This trend suggests that the stabilization effect is related, among other factors, to the dielectric constant of the alcohols. Surprisingly, even though the gelation was only observed at 5 °C with the three alcohols, the T_gel_ were always higher and in the case of the methanolic gel it was stable even at room temperature.

**Table 1:** Gelation tests of triphenylene derivatives **1**–**6**.

Entry	Solvent	1	2	3	4	5	6
1	*n*-Hexane	I	I	I	I	I	G (0.25)
2	Toluene	S	S	S	S	S	G (0.35)
3	Dichloromethane	S	S	S	S	S	S
4	Ethylether	S	S	S	S	S	S
5	Ethylacetate	S	S	S	S	S	S
6	Methanol	TG * (0.1)	TG * (0.2)	TG * (0.2)	I	TG * (0.5)	TG * (1.0)
7	Water	I	I	I	I	I	I

Gelation test: * at 5 °C; G: Gel; TG: Turbid gel; S: Soluble; I: Insoluble; in brackets: Critical concentration for gelation (wt %).

**Table 2:** Gel-sol transition temperatures for gels of acid **2** (1 wt %) in three different alcohols and their dielectric constants.

Entry	Solvent	T_gel_ (°C)	*ε* *
1	Methanol	31	33.6
2	Ethanol	17	25.2
3	*n*-Butanol	8	18.2

* Dielectric constants at 20 °C [24].

A totally different behavior was found for the triphenylene amines **4**–**6**; while compound **4** with the shortest spacer did not gel any solvent, amine **6** with the longer spacer was able to gel hydrocarbons such as *n*-hexane and toluene even at room temperature to give transparent, stable gels. Triphenylene amine **5** with the spacer length that leads the functionality on the edge of the hydrophobic corona only gelled small alcohols at 5 °C, in a similar way as acids **1**–**3**.

**Figure 2:**
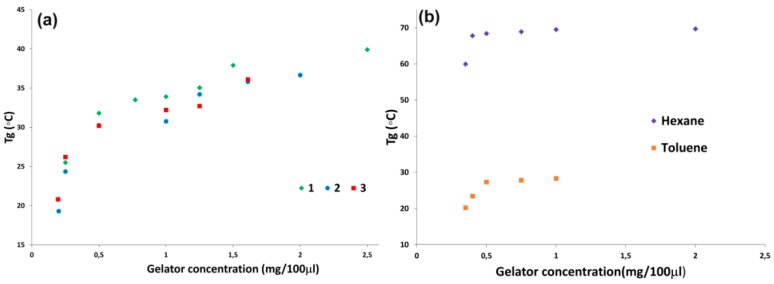
T_gel_ *vs.* concentration plots as a measure of thermal stability. (**a**) Methanol gels based on triphenylenes **1**, **2**, and **3**; and (**b**) hydrocarbon gels based on triphenylene amine **6**.

To estimate the thermal stability of the gels we studied the variation of the gel-sol transition temperature (T_gel_) with the concentration of gelator for acids **1**–**3** in methanol (Figure 2a) and in *n*-hexane and toluene for amine **6** (Figure 2b). Tube inversion experiments were performed to measure T_gel_, this method was selected because of its simplicity and widespread use in the field of gel-phase materials. In all cases as the concentration of gelator increased, T_gel_ also increased until a plateau region was reached at about the same concentration for the three acids (1.5 wt %). Near the critical concentration for gelation (CCG) the methanolic gels of **1**–**3** were almost transparent but, as the concentration increased, they became turbid, accounting for partial precipitation and low solubility. At low concentration of gelator the T_gel_ *vs.* concentration profile for the three acids is almost the same. For concentrations above 1.5 wt % the acid with the shortest linker (**1**) shows a slightly higher stability with a T_gel_ of 40 °C. The *n*-hexane gel of amine **6** has the higher thermo stability with a maximum T_gel_ of 70 °C reached at a low concentration near the CCG (0.5 mg/100 μL, Figure 2b). In aromatic hydrocarbons, such as toluene, of higher polarity compared to *n*-hexane, the maximum T_gel_ decreases to 28 °C. From these experiments we can conclude that the amine derivatives **4**–**6** have a very different gelation scope than the carboxylic acid derivatives since in these solvents (*n*-hexane and toluene) hydrogen bond interaction with the polar amine heads is not possible.

### 2.2. Morphology and Self-Assembly

**Figure 3:**
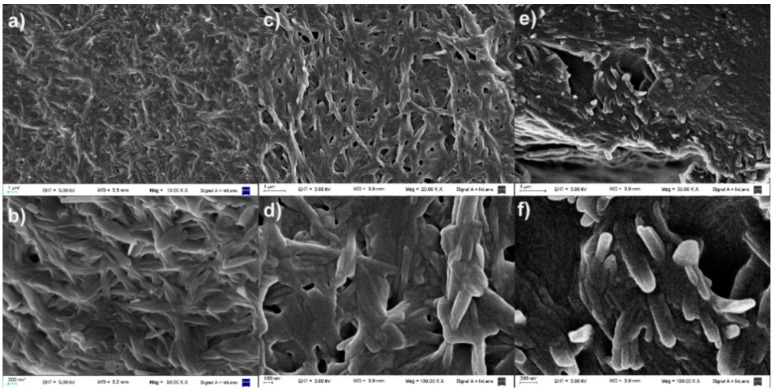
SEM images of xerogels from methanol of acids **1** (**a,** Mag = 10.00 KX), (**b**, Mag = 50.00 KX), **2** (**c**, Mag = 25.00 KX), (**d**, Mag = 100.00 KX), and **3** (**e**, Mag = 30.00 KX), (**f**, Mag = 100.00).

To obtain an insight into the microstructures of the gels we analyzed the xerogels of triphenylenes **1**–**3** and **5**–**6** by SEM microscopy (Figure 3). The images showed a typical entangled fibrillar network for all the xerogels. The observed widths of the fibers are wider than the theoretical average molecular length of 2.55 nm (estimated by computational simulation, AM1) suggesting that the observed structures are composed of several bundles of one-dimensional stacks of 90–180 nm. In case of the amine derived xerogels, which rendered a transparent sticky glassy material after evaporation of the solvent [23], the SEM images showed a diffused, less defined fibrillar network due to the collapse of the fibrils after evaporation of the solvent (see Figure S1 in Supplementary Information).

**Figure 4:**
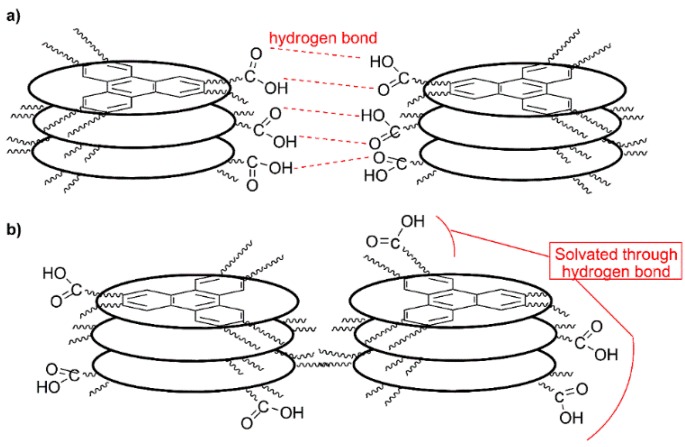
Possible self-assembly models for gels of triphenylene **1**–**3** in alcohols. (**a**) Hydrogen bond dimerization between the carboxylic functionalities, disfavored due to solvophobic effects; and (**b**) proposed mode of self-assembly with stabilization of the 1D columnar packing through solvation of the carboxylic groups located in the periphery pointing out to the polar environment (alcohol).

Shimizu and co-workers [25] have studied a homologous series of 1,2-bifunctionalized triphenylene derivatives bearing two carboxylic acids at the end of adjacent aliphatic chains. They demonstrated, using infrared spectroscopy, that in bulk they show a columnar mesophase with a dimeric superstructure caused by the intermolecular hydrogen bonding interaction between the carboxylic groups (Figure 4a). Nevertheless, this mode of self-assembly is rather unlikely for the alcoholic gels since the COOH function would be in a non-polar environment. We propose that for gels of acids **1**–**3** in alcohols the 1-D self-assembly should be directed by π-π stacking between the triphenylene cores with complementary stabilization by solvation of the polar COOH groups located in the periphery through strong hydrogen bonds with the solvent (Figure 4b). This mode of assembly is in agreement with the fact that, as the dielectric constant of the alcohols increases, so does the thermal stability of the gels (Table 2).

**Figure 5:**
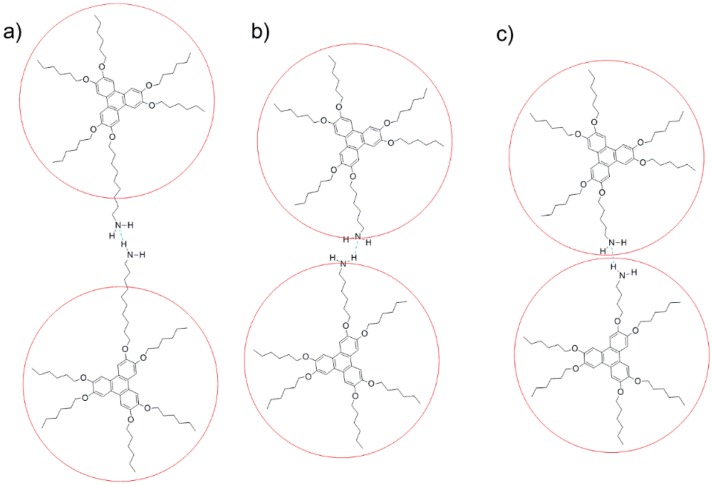
Schematic representation for the self-assembly of triphenylene amines through hydrogen bond (dashed blue line). (**a**) Amine **6**, hydrogen bond was observed in FT-IR studies; (**b**) amine **5**, the amine group is on the edge of the alkyl corona (in red) and (**c**) amine **4**, in this case hydrogen bond dimerization is disfavored due to steric hindrance.

The gels of amine **6** in hydrocarbons such as *n*-hexane and toluene are a completely different system. In these cases solvation of the NH_2_ group through hydrogen bond is not possible, and the amine group of one molecule should be involved in an intermolecular hydrogen bond with another gelator molecule (Figure 5). This was confirmed by FT-IR experiments of a dichloromethane (DCM) solution and *n*-hexane gel of **6**, which are both hydrogen bond inactive solvents. In the DCM solution, a sharp band was observed at *ν*_N-H_ = 3678 cm^−1^ typical for free amines. In the gel state this band was broadened and shifted to 3448 cm^−1^, accounting for the presence of hydrogen-bond associated N–H (see Figure S2 in Supplementary Information). As only amine **6** is capable of gelling hydrocarbons, it seems to be essential for the amine group to be outside the alkyl corona to allow the intermolecular hydrogen bond interactions between the amine functions (Figure 5a). This is supported by the fact that compound **4**, with the amine group inside the alkyl corona, cannot efficiently interact through intermolecular hydrogen bonds due to steric hindrance (Figure 5c) and cannot gel any solvent. Nevertheless triphenylene **5** with the amine group on the edge of the alkyl corona is a border case. For this compound formation of hydrogen bonded dimers is still possible since the amine group is just on the edge as can be seen on Figure 5b, but this compound can only gel alcohols. This indicates that, at least for amine **5**, a subtle balance between gelator-gelator and solvent-gelator interactions is crucial and the ability to form hydrogen bonded dimers is not the only aspect to be considered.

**Figure 6:**
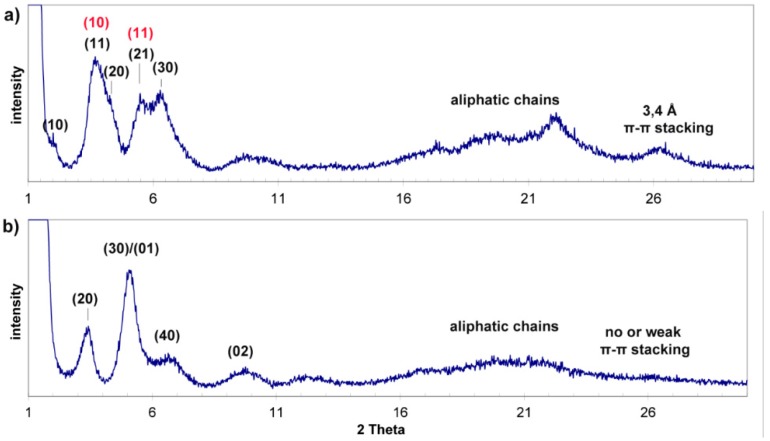
X-ray diffraction patterns of: (**a**) Xerogel of triphenylene **5** from methanol (in red, reflections of the tetragonal array overlapped with the (11) and (21) reflections of the hexagonal lattice) and (**b**) xerogel of triphenylene **6** from *n*-hexane.

**Figure 7:**
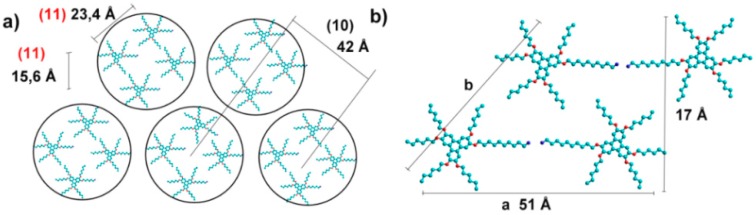
Proposed self-assembly for gels of triphenylene **5** in methanol (**a**) and triphenylene **6** in *n*-hexane (**b**). Structures: blue (carbon), red (oxygen), and violet (nitrogen).

In order to assess the supramolecular organization of the functionalized triphenylenes in the gel state we performed both WAXS and SAXS experiments (*T* = 5 °C) on samples of gel of **5** in MeOH and gel of **6** in *n*-hexane. Unfortunately, no clear information could be obtained from these experiments since the gels are diluted systems and the solvent hardly interferes. We thus turned our attention to the xerogels resulting from these mixtures assuming this organization could closely resemble that of the gels and performed powder XRD experiments on these dried samples. The results are shown in Figure 6. The XRD pattern of the xerogel of **5** from methanol could be indexed in a simple hexagonal 2-D system with an intercolumnar distance of 49 Å; reflections (10), (11), (20), (21), and (30) were seen, as shown in Figure 6a. Additionally, broad peaks were also detected at ca. 4.5 Å and 3.6 Å, corresponding to methylene-methylene and π-π distances, respectively. The intercolumnar distance is high for a simple stacking of triphenylene units in columns self-organized into a hexagonal array, where values close to 20–25 Å are expected. Moreover, the intensities of the peaks observed at 3.78 and 5.60° are too high for (11) and (21) reflections. We thus suggest that each column of the hexagonal array is built up by four columns of individual triphenylene units arranged in a tetragonal system, held together by π-π interactions and van der Waals interactions between aliphatic chains (Figure 7a). The (10) and (11) reflections of this tetragonal array (*a* = 23 Å) overlapped with those of the (11) and (21) reflections of the hexagonal lattice, explaining their high intensity. These supramolecular columnar arrays are likely to be present in the gel state with a high percent of NH_2_ groups pointing toward the periphery, interacting with the solvent (methanol).

The XRD pattern of the xerogel of triphenylene **6** from *n*-hexane was slightly different: at wide angles no peak related to π-π interactions could be firmly discerned and at small angles the peaks could be successfully indexed either in a 2-D-rectangular or a 2-D-oblique system (see Figure 6b for indexation). In the first case, the resulting cell parameters would be *a* = 51 Å and *b* = 17 Å; in the second case, these distances would correspond to the side and the height of a parallelogram respectively (Figure 7b). We think the second choice allows for a better interpretation in terms of supramolecular arrangements; indeed, it is fully consistent with a situation in which the polar terminal NH_2_ groups are “hidden” to the solvent, being located at the center of a supramolecular H-bonded dimer. The expected length of such a dimer (estimated as 60 Å if fully extended chains are assumed) is consistent with the measured 51 Å value. Similar distances have been found in the liquid crystalline phase of hybrid POSS-triphenylene compounds [26] for which related molecular conformation was suggested (in this case POSS acts as a covalent linker between the triphenylene cores in analogy to the amine hydrogen bonds on the dimeric assembly of gelator **6**).

Fluorescence spectroscopy can provide relevant information about the molecular self-organization of fluorophores, in this case the triphenylene core. As already shown in Table 1, all gels were turbid with exception of the *n*-hexane gel of amine **6** witch exhibited excellent transparency for the optical characterization. Emission spectra of the *n*-hexane gel and a chloroform solution of **6** were obtained by excitation at 350 nm. A shift to longer wavelengths was observed in gel state (see Figure S3 in Supplementary Information). A similar red-shift is typically observed in the liquid crystalline (LC) phase of triphenylene derivatives due to a stabilization effect on the excited state in the aggregates [18]. These results indicate that the molecular organization on the *n*-hexane gel is related to the conventional staggered (or helical) overlap between the triphenylenes cores usually found in LC phase.

### 2.3. Effect of pH on Gelation

**Figure 8:**
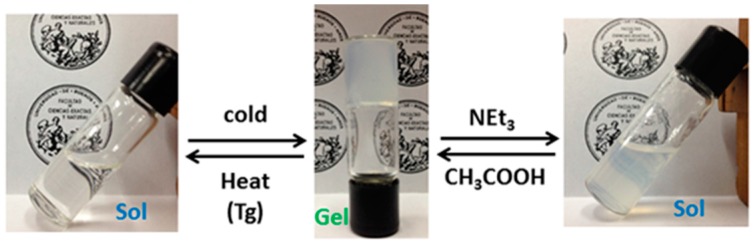
Thermic and pH triggered sol-to-gel and gel-to-sol transitions of organogelator **3** in methanol.

The presence of carboxylic acid and amine groups in organogelators **1**–**6** and their role on the stabilization of the fibrillar matrix prompted us to study the effect of a change of pH on the gelation ability. Addition of a mild base such as triethylamine to the methanolic gels of acids **1**–**3** at 5 °C promoted the gel-to-sol transition due to a disruption of the supramolecular self-assembly caused by partial deprotonation of the carboxylic groups (Figure 8). This transition could be seen to the naked eye by inversion of the tube. The process was reversible and neutralization with acetic acid regenerated the gel at the same temperature. The experiment was also performed on the methanolic gels of amines **5** and **6** but after addition of acetic acid only a partial gel-to-sol process was observed that could be reversed by addition of triethylamine. The *n*-hexane gel of amine **6** did not respond to the addition of acetic acid, probably due to a slow diffusion into the non-polar gel matrix. We also tested the gelation ability of a 1:1 mixture of amine **5** and acid **2** on alcohols at different final concentrations. In any case formation of an ammonium carboxylate salt with very low solubility was evident from precipitation of a crystalline white solid that did not form gel after a heating-cooling process. From these results we can conclude that the presence of a negative or positive charge on the acid and amine triphenylene derivatives inhibits the gelation ability and disrupts the self-assembly network in a reversible way.

### 2.4. Self-Catalyzed Template Synthesis

**Figure 9:**
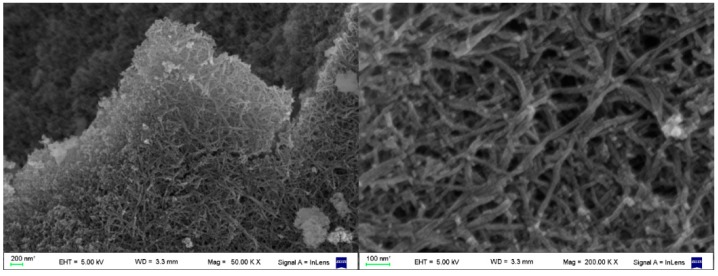
SEM images of the nanotubular entangled network of silica obtained by *in situ* polymerization of TEOS using as template a gel of **5** in methanol, free of added catalyst.

The *in situ* sol-gel polymerization of inorganic alcoxides in supramolecular gels has been applied with success in the preparation of nanostructured materials based on inorganic oxides (TiO_2_, SiO_2_, *etc.*) with defined morphology and potential application in nanotechnology [27]. The template process is more effective if a positive charge is present in the gelator structure to guide the growing negative charged silicate intermediaries through electrostatic interactions [28], but in some cases template can be achieved with neutral gelators through hydrogen bond or dipolar interactions [29]. Additionally, the potential application of such nanostructured inorganic materials, the template polymerization process can be used to study the morphology of the self-assembled fibrillar network (SAFIN) of the gel since the nanostructured material obtained is a direct copy of the superstructure network present in gel phase that, as mentioned before, collapses after evaporation of the solvent (xerogel). Since the hydrolytic polymerization of TEOS is usually performed in the presence of acid and/or basic catalysis (usually benzyl amine), it was our goal to study the feasibility of using the carboxylic and amine groups present in the gels as catalytic centers on the SAFIN surface. Templated polymerization experiments with titanium isopropoxide on methanolic gels were infructuous because addition of the titanium alcoxide avoided formation of the gels rendering transparent solutions, probably caused by a strong interaction with the amine and carboxylic groups of the triphenylene derivatives. Nevertheless the gels of triphenylenes **2**, **3**, and **5** in methanol were stable to the addition of TEOS (5% *v*/*v*) and water (0.05% *v*/*v*) and after 10 days of at 5 °C the silica obtained was analyzed by SEM microscopy. With gels of triphenylenes **2** and **3** the silica obtained was highly amorphous (see Figure S4 in Supplementary Information), but in case of amine **5** the SEM images showed an entangled fibrillar network of silica nanotubes with an external diameter of 20–25 nm (Figure 9). The morphology observed is a direct evidence of the fibrillar nature of the self-assembled network present on the gel.

## 3. Conclusions

In summary, we have studied the supramolecular organogelling properties of six asymmetrical hexaether derivatives of triphenylene mono-functionalized with carboxylic and primary amine groups. The acid derivatives **1**–**3** were able to selectively gel small alcohols independently of the length of the linker, suggesting an assembly where the columnar discotic array is stabilized by solvation of the carboxylic groups, in an analog way as proposed for Kotlewski *et al.* [20] for related mono-substituted triphenylenes π-gelator bearing a primary alcohol functionality. In case of the amine derivatives **4**–**6** the linker length was a determining factor for the gelling ability. Triphenylene **4** with the amine group inside the alkyl corona was not able to gel any of the solvents while amine **5** and **6** gelled small alcohols. Particularly amine **6** with the longest linker was able to gel hydrocarbons at room temperature and FT-IR experiments revealed that intermolecular hydrogen bonds between amine groups are involved in the self-assembly that leads to the SAFIN. This, together with XRD experiments allowed us to propose a self-assembly mode in which the polar terminal NH_2_ groups are “hidden” to the solvent, being located at the center of a supramolecular H-bonded dimer consistent with the molecular dimensions. All methanol derived gels were responsive to temperature and pH changes in a reversible way, but the hydrocarbon derived gels of **6** were stable to addition of acid probably by the fact that the amine function is protected by the nonpolar surrounding that avoids diffusion of the acid. The methanolic gel of **5** was successfully used as template for the preparation of silica nanotubes were templation was directed by a self-catalytic process. The template polymerization allowed a direct view of the real, fibrillar structure of the SAFIN on the gel that was not observable from the SEM images of the collapsed xerogel. In conclusion, the presence of a carboxylic and amine group on the triphenylene core generates pH/temperature responsive π-organogels and these results can be used to design new pH-responsive π-gelators with specific electronic properties. In view of the interesting properties of the gels derived from these simple mono-functionalized triphenylenes a further study of symmetric and asymmetric bi-functionalized triphenylenes derivatives is now in progress.

## 4. Experimental Section

### 4.1. Materials and Methods

IR spectra of solid samples were recorded in thin films using KBr disks on a Thermo Scientific™ Nicolet™ iS50 FT-IR (Thermo Scientific, Waltham, MA, USA). ^1^H and ^13^C-NMR spectra were measured in a Bruker Avance II 500 (500.13 and 125.72 MHz) NMR spectrometer (Bruker Daltonics Inc., Billerica, MA, USA), in deuteriochloroform using TMS as internal standard. Exact mass spectra (ESI) were measured on an Agilent LCTOF, high resolution TOF analyzer with APCI/ESI ionization (Agilent Technologies, Santa Clara, CA, USA). MALDI-TOF spectra were recorded on a Bruker Daltonics OmniFlex apparatus in dithranol matrix (Bruker Daltonics Inc., Billerica, MA, USA). Elemental analysis was performed on an EAI Exeter CE-440 apparatus (Exeter Analytical Inc., Coventry, UK). XRD experiments were performed at room temperature on a Siemens D5000 diffractometer (Siemens AG, Munich, Germany), using the K-α copper radiation from a curved-graphite monochromator and planar glass sample holders. Tetraethyl orthosilicate (TEOS, >99.0%, Fluka Analytical) and Titanium(IV) isopropoxide (purum, Aldrich) were used as received. Triphenylene carboxylic acids **2** [21] and **3** [22] and amines **4**–**6** [23] were synthesized following reported experimental procedures and their spectroscopic data were identical to those found in the literature.

4-(3′,6′,7′,10′,11′-Pentahexyloxytriphenylen-2′-yloxy)butanoic acid (**1**) was prepared following a reported methodology [22]: To a mixture of 3,6,7,10,11-pentahexyloxy-2-hydroxytriphenylene (1.5 mmol), tetraethylammonium iodide (54 mg) and anhydrous potassium carbonate (2.6 g) in CH_3_CN (15 mL) was added a solution of ethyl,4-clorobutanoate (1.8 mmol ) dissolved in CH_3_CN (6 mL). The mixture was refluxed for 20 h, then cooled to room temperature and poured into CHCl_3_ (50 mL). The solids were filtered off, the organic layer was dried with Na_2_SO_4_ and the solvent was evaporated *in vacuo*. The resulting syrup was suspended in EtOH (30 mL) and a solution of sodium hydroxide (0.48 g) in water (3 mL) was added under argon atmosphere and then refluxed for 4 h. The cooled mixture was carefully poured into an aqueous solution of hydrochloric acid (2 M). The white precipitated was extracted with chloroform (2 × 50 mL). The organic layer was dried and the solvent evaporated. The crude product was purified by column chromatography on silicagel eluting with cyclohexane-ethyl acetate (50:50 → 30:70) to give the triphenylene mono acid **1** (78.5% yield). FT-IR (KBr, cm^−1^) ν 3268 (broad); 2926; 2856; 1718; 1617; 1518; 1438; 1261.

^1^H-NMR (500 MHz, CDCl_3_) δ (ppm) 7.87 (d, 6H), 4.33 (t, 2H), 4.26 (t, 10H), 2.76 (t, 2H), 2.30 (m, 2H), 1.96 (m, 10H), 1.48 (m, 30H), 0.96 (t, 15H). ^1^H-NMR spectrum can be found in the Supplementary Information File, Figure S5.

^13^C-NMR (125 MHz, CDCl_3_) δ (ppm) 176.6; 149.1; 149.0; 149.0; 148.95; 148.9; 148.4; 124.0; 123.7; 123.6; 123.5; 123.5; 107.7; 107.4; 107.3; 107.2; 107.1; 107.0; 76.9; 76.8; 69.8; 69.7; 69.7; 69.5; 68.5; 31.7; 31.7; 30.3; 29.4; 29.3; 25.9; 24.6; 22.7; 22.7; 14.1. ^13^C-NMR spectrum can be found in the Supplementary Information File, Figure S6. HRMS (ESI) calcd for C_52_H_78_NaO_8_ (M + Na^+^) 853.5589; found 853.5589.

*11-(3′,6′,7′,10′,11′-Pentahexyloxytriphenylen-2′-yloxy)undecanoic acid* (**3**) To a mixture of 3,6,7,10,11-pentahexyloxy-2-hydroxytriphenylene (1.5 mmol), tetraethylammonium iodide (54 mg) and anhydrous potassium carbonate (2.6 g) in CH_3_CN (15 mL) was added a solution of ethyl 11-bromoundecanoate (1.8 mmol ) in CH_3_CN (6 mL). The mixture was refluxed for 20 h, then cooled to room temperature and poured into CHCl_3_ (50 mL). The solids were filtered off, the organic layer was dried with Na_2_SO_4_ and the solvent was removed *in vacuo*. The resulting syrup was suspended in EtOH (30 mL) and a solution of sodium hydroxide (0.48 g) in water (3 mL) was added under argon atmosphere and was refluxed for 4 h. The cooled mixture was carefully poured into an aqueous solution of hydrochloric acid (2 M). The white solid was extracted with chloroform (2 × 50 mL). The organic layer was dried and the solvent evaporated. The crude product was purified by column chromatography on silica eluting with cyclohexane-ethyl acetate-acetic acid (90:10:0.05 → 80:20:0.05) to give the triphenylene mono acid **3** (Yield 69%).

^1^H-NMR (500 MHz, CDCl_3_) δ (ppm) 7.83 (s, 6H), 4.22 (t, 12H), 2.34 (t, 2H), 1.96–1.90 (m, 12H), 1.65–1.53 (m, 14H), 1.44–1.32 (m, 30H), 0.93 (t, 15H).

^13^C-NMR (125 MHz, CDCl_3_) δ (ppm) 177.62; 148.95; 123.59; 107.33; 69.69; 33.81; 31.67; 29.56; 29.46; 29.31; 29.24; 29.04; 26.16; 25.83; 22.64; 14.04 Anal. Calcd for C_59_H_92_O_8_: C, 76.2; H, 10.0. Found: C, 76.1; H, 9.7. MALDI-TOF: *m*/*z* 928.71 ([M]^+^); 929.71 ([M + 1]^+^); 930.71 ([M + 2]^+^); 931.70 ([M + 3]^+^).

### 4.2. Gelation Test

The tests were carried out in a similar manner as described in a previous work [29]. The gelation ability was investigated by a typical test tube experiment. A mixture of a defined amount of gelator and a volume of the solvent (10% wt/v) in a closed flask was heated and shaken until the solid was dissolved and then slowly cooled to room temperature. If a stable gel was observed after inversion of the flask, it was considered a gel (G). When gelation was not observed at room temperature, the sample was cooled at 5 °C. The critical concentration for gelation (CCG) was determined by subsequent dilution of the original organogel followed by a heating-cooling process until gel formation was not observed. The reversible gel-sol transition temperatures (*T*_gel_) were measured using the classical inverted tube method [30].

### 4.3. FT-IR Spectra

The measurements were carried in a similar manner as described in [29]. Fourier transform infrared (FTIR) measurements of the solution and gel of **6** were performed on Thermo Scientific™ Nicolet™ iS50 FT-IR (Thermo Scientific, Waltham, MA, USA) spectrometer in a demountable liquid cell with two NaBr disks, 32 mm in diameter and a 0.5 mm thick Teflon spacer. For the *n*-hexane gel, a warm solution of **6** (0.25%) was injected into the cell and allowed to cool down for 10 min at room temperature before measuring the spectra.

### 4.4. Xerogel Preparation

The xerogels were prepared by cooling the gels in a bath at −10 °C, evaporating the solvent under high vacuum for 6 h and then slowly letting the gels cool to room temperature under vacuum.

### 4.5. SEM Microscopy

SEM pictures of the xerogel and silica nanoparticles were taken on a Carl Zeiss NTS SUPRA 40FEG scanning electron microscope (Carl Zeiss GA, Munich, Germany). A small portion of the solid sample (xerogel or silica) was attached to the holder by using a conductive adhesive carbon tape. Prior to examination the xerogels were coated with a thin layer of gold.

### 4.6. In Situ Sol-Gel Polymerization Experiments

General procedure: A mixture of organogelator (10 mg) and methanol (0.95 mL) was heated in a closed flask until total dissolution, subsequently the inorganic alcoxide (Ti(*i*-OPr)_4_ or TEOS, 50 μL) and water (6 μL) were added. The solution was cooled on the fridge at 5 °C until gelation was observed and then left for 10 days. The sample was dissolved in dichloromethane, the solid was centrifugated, and washed once with dichloromethane. The inorganic oxide so obtained was heated at 200 °C for 2 h and 500 °C for 4 h in air.

### 4.7. Fluorescence Experiments

Corrected fluorescence emission spectra were obtained on a Cary Eclipse spectrophotometer (Agilent Technologies, Santa Clara, CA, USA) equipped with two Czerny-Turner monochromators and a 15 W Xenon pulse lamp (pulse width: 2–3 us, power: 60–75 kW), the probe was excited at 350 nm. The fluorescence measurements were conducted using the right angle technique. For the determination of the emission spectra of the gel a mixture of the gelator and solvent (0.25 wt %) was heated (80 °C) and shaken in a closed flask until the gelator was dissolved. Then, the warm solution was placed in a cuvette and cooled to room temperature to form a stable transparent gel before acquiring the emission spectrum.

## Supplementary Materials

Supplementary File 1
