# Supplementary material for: Supramolecular Assembly of pH-Sensitive Triphenylene Derived π-Gelators and Their Application as Molecular Template for the Preparation of Silica Nanotubes
Source: Gels. 2016 Feb 1;2(1):7. doi: 10.3390/gels2010007 (PMC6318639; doi:10.3390/gels2010007)
Supplement: Supplementary File 1 [file gels-02-00007-s001.pdf]

Supplementary Materials: Supramolecular Assembly of pH Sensitive Triphenylene Derived π-Gelators and Their Application as Molecular Template for the Preparation of Silica Nanotubes

Ignacio Muñoz Resta, Verónica E. Manzano, Florencia Cecchi, Carla C. Spagnuolo, Fabio D. Cukiernik and Pablo H. Di Chenna

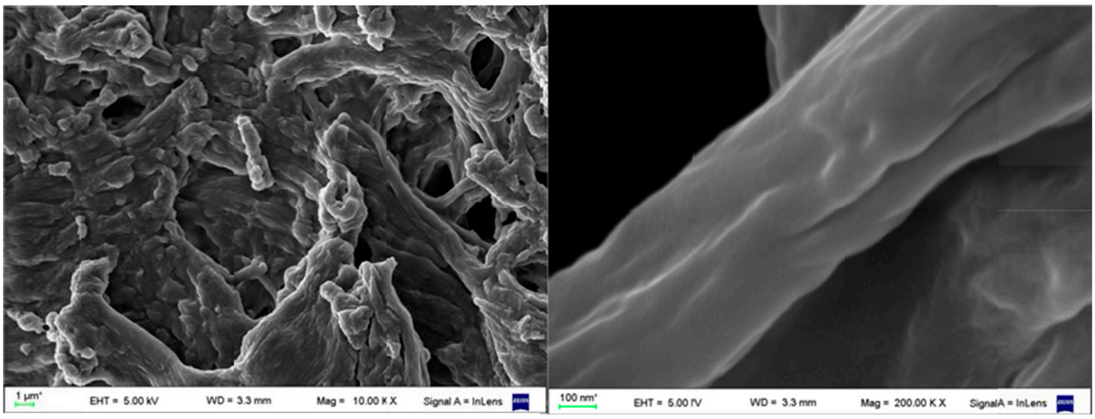

Figure S1. SEM images of Xerogel of amine derivative **5** from methanol.

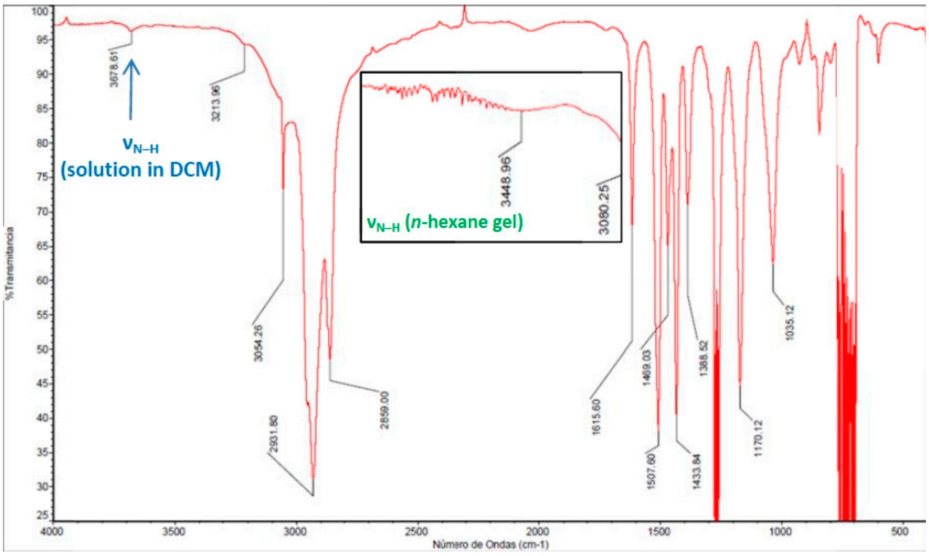

Figure S2. FT-IR spectra of compound **6** (**a**) solution in DCM (**b**) gel in *n*-hexane.

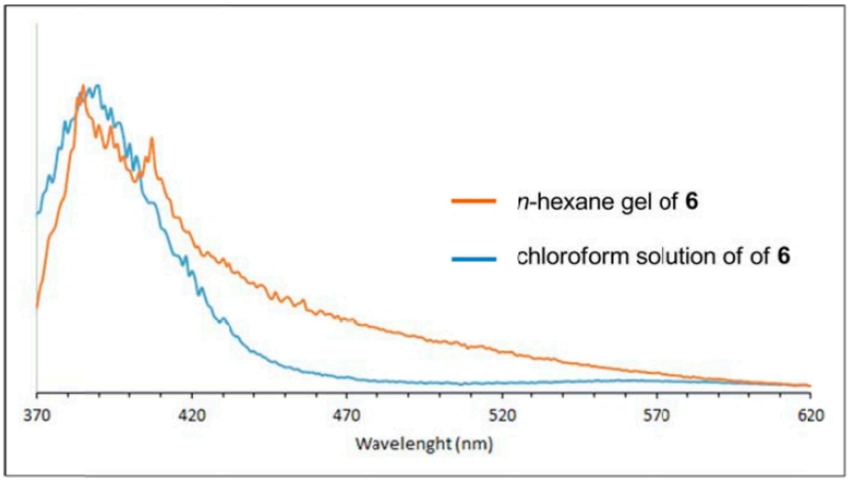

Figure S3. Fluorescence spectra of a chloroform solution and *n*-hexane gel of amine **6** (concentration 0.25%wt., λ_{exc} 350 nm, Temperature: 24 °C).

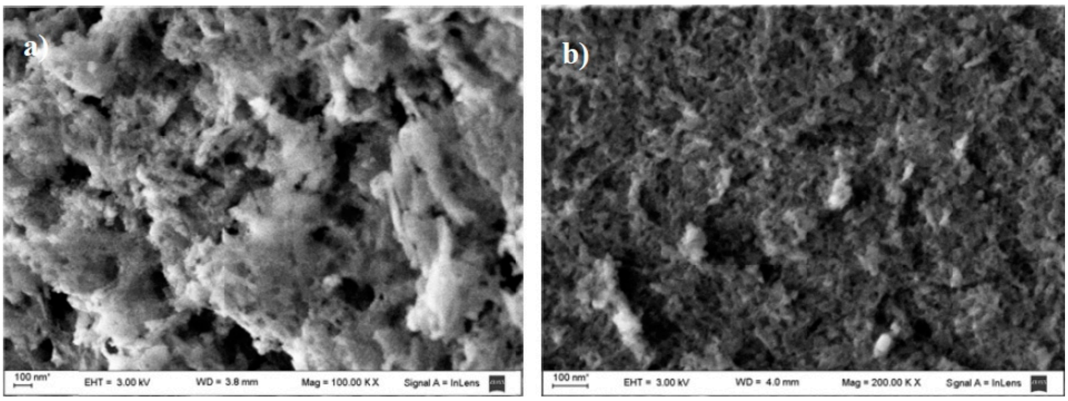

Figure S4. SEM images of amorphous silica obtained by *in situ* sol-gel polymerization from: (**a**) acid **2** (**b**) acid **3**.

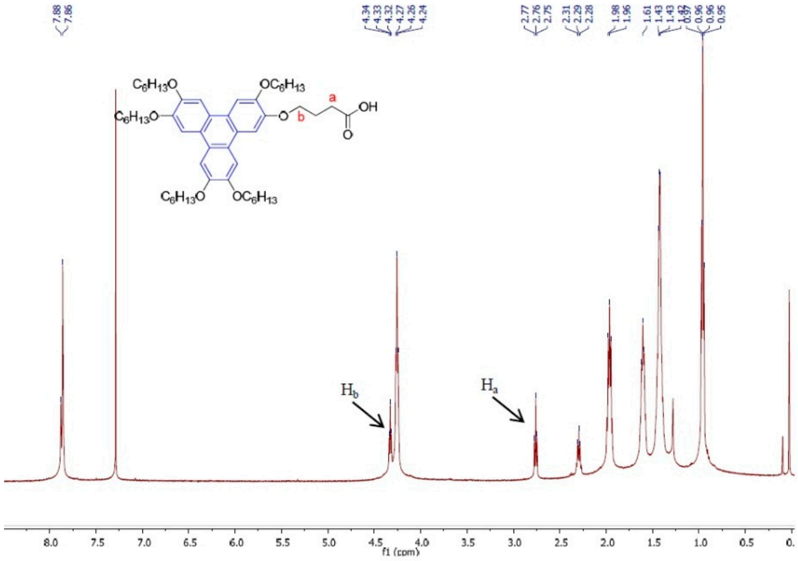

Figure S5. ^{1}H-NMR (500 MHz, $CDCl_3$) of triphenylene 1.

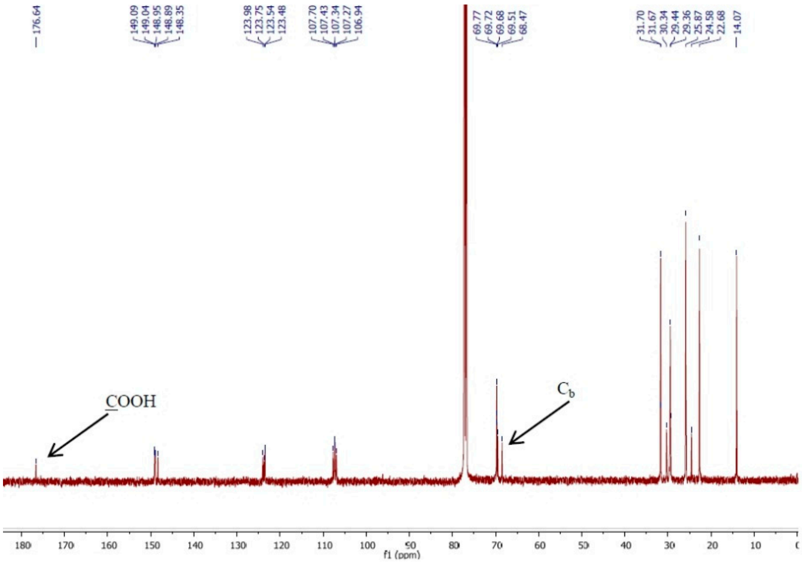

Figure S6. ^{13}C-NMR (125 MHz, $CDCl_3$) of triphenylene 1.

© 2016 by the authors; licensee MDPI, Basel, Switzerland. This article is an open access article distributed under the terms and conditions of the Creative Commons by Attribution (CC-BY) license (http://creativecommons.org/licenses/by/4.0/).
